# Philip S. Dorland, M.D.

**Published:** 1867-05

**Authors:** 


### OBITUARY.

---

Died—August 15, 1866, in Chicago, Philip S. Dorland, M. D., of East Hamburgh, Erie county, N. Y.

Our departed brother was worthy of something more than just the passing notice, "died." He left his field of usefulness in early manhood, being but twenty-nine years old at his death, yet he had won for himself a reputation for professional skill, for christian kindness and sympathy, for strict integity, and for faithfulness in the discharge of every duty that many of the older members of the fraternity might well envy. His success was achieved by his own unaided efforts. He was emphatically a worker. He ever felt that he was engaged in a very serious and very responsible business, and upon his death-bed in looking over his past career in the light of eternity, he declared that in every case of sickness entrusted to his care, he believed he had done his whole duty to the best of his ability, and he expressed joy at the prospect of being translated through the goodness of his Heavenly Father from this scene of labor and responsibility to those realms of blessedness that he felt he was going to inhabit. Faithful and true in life, he was cheerful and happy in death. May his short but successful career be the means of stimulating the young men of our profession to increased diligence and honesty, the only sure road to permanent success.

The immediate cause of his death was hemorrhage from the lungs. The great exposure inevitably connected with an extensive country practice in this latitude had produced a severe bronchitis from which he had suffered much for two or three years previous to his death; occasional spitting of blood accompanying it. He was on his way to Minnesota for the benefit of his health, and had reached Chicago in good spirits and full of hope, but was there prostrated by a very severe attack of hæmorrahage, from which he was unable to rally. He had the benefit of the skill and unremitting attention of that excellent physician and christian gentleman, Prof. Davis, of Chicago. He survived the attack but a few days, passing away at peace with all the world. His remains were brought to East Hamburgh for interment, where an immense concourse of people testified their love of the man and appreciation of his worth by their sadness, their expressions and their tears. Peace to his ashes.

East Hamburgh, May 11, 1867.

